# Work Conditions And Life Satisfaction Among Older American Adults: The Moderating Role Of Marital Status

**DOI:** 10.1093/geroni/igaf122.2347

**Published:** 2025-12-31

**Authors:** Kingsley Mbam, Jeffrey Burr

**Affiliations:** University of Massachusetts Boston, Boston, Massachusetts, United States; University of Massachusetts Boston, Boston, Massachusetts, United States

## Abstract

Although past studies have explored the connection between work conditions and life satisfaction among older adults, whether this association varies by marital status is largely unaddressed. This study aimed to examine the relationship between work conditions and life satisfaction among older American adults and to investigate whether marital status moderates this association. This study analyzed models of life satisfaction utilizing data from the 2020 wave of the Health and Retirement Study for respondents aged 55-75 (N = 937). Linear regression models were utilized to analyze the association between a variety of subjective work conditions and life satisfaction and whether marital status conditions this association. Life satisfaction was assessed utilizing Diener’s measure of life satisfaction. Marital status was measured as follows: 1=married, 0=not married. The results of the linear regression models showed that some work conditions - better mood at work due to family or personal life (b = 3.81, p = 0.000), better mood at home due to job (b = 2.94, p = 0.000), work gives energy to do things with family and other people (b = 2.86, p = 0.000), and job worries distraction when not at work (b=-4.03, p = 0.000) - are significantly related to life satisfaction, controlling for relevant covariates. The results also revealed that the interaction of job worries distraction when not at work and married for life satisfaction was statistically significant (b = 3.35, p = 0.04). These results suggest that fostering supportive and favorable working conditions for older workers can enhance their life satisfaction, which is important to improving their overall health and well-being.

